# Non-Thermal Plasma Reduction of Ag^+^ Ions into Silver Nanoparticles in Open Atmosphere under Statistically Optimized Conditions for Biological and Photocatalytic Applications

**DOI:** 10.3390/ma15113826

**Published:** 2022-05-27

**Authors:** Noor Ul Huda Altaf, Muhammad Yasin Naz, Shazia Shukrullah, Madiha Ghamkhar, Muhammad Irfan, Saifur Rahman, Tomasz Jakubowski, Esam A. Alqurashi, Adam Glowacz, Mater H. Mahnashi

**Affiliations:** 1 Department of Physics, University of Agriculture Faisalabad, Faisalabad 38040, Pakistan; yasin306@uaf.edu.pk; 2 Department of Mathematics and Statistics, University of Agriculture Faisalabad, Faisalabad 38000, Pakistan; madiha_gm@yahoo.com; 3 Electrical Engineering Department, College of Engineering, Najran University Saudi Arabia, Najran 11001, Saudi Arabia; miditta@nu.edu.sa (M.I.); srrahman@nu.edu.sa (S.R.); 4 Faculty of Production and Power Engineering, University of Agriculture in Krakow, Balicka 116 B Str., 30-149 Krakow, Poland; tomasz.jakubowski@urk.edu.pl; 5 Department of Chemistry, Faculty of Science, University of Albaha, Albaha 1988, Saudi Arabia; ealqurashi@bu.edu.sa; 6 Department of Electrical Engineering, Cracow University of Technology, Warszawska 24 Str., 31-155 Krakow, Poland; adglow@agh.edu.pl; 7 Department of Pharmaceutical Chemistry, College of Pharmacy, Najran University, Najran 61441, Saudi Arabia; mhmahneshi@nu.edu.sa

**Keywords:** plasma reduction reaction, silver nanoparticles, response surface methodology, wastewater treatment, antioxidant activity

## Abstract

An environmentally friendly non-thermal DC plasma reduction route was adopted to reduce Ag^+^ ions at the plasma–liquid interface into silver nanoparticles (AgNPs) under statistically optimized conditions for biological and photocatalytic applications. The efficiency and reactivity of AgNPs were improved by statistically optimizing the reaction parameters with a Box–Behnken Design (BBD). The size of the AgNPs was chosen as a statistical response parameter, while the concentration of the stabilizer, the concentration of the silver salt, and the plasma reaction time were chosen as independent factors. The optimized parameters for the plasma production of AgNPs were estimated using a response surface methodology and a significant model *p* < 0.05. The AgNPs, prepared under optimized conditions, were characterized and then tested for their antibacterial, antioxidant, and photocatalytic potentials. The optimal conditions for these three activities were 3 mM of stabilizing agent, 5 mM of AgNO_3_, and 30 min of reaction time. Having particles size of 19 to 37 nm under optimized conditions, the AgNPs revealed a 82.3% degradation of methyl orange dye under UV light irradiation. The antibacterial response of the optimized AgNPs against *S. aureus* and *E. coli* strains revealed inhabitation zones of 15 mm and 12 mm, respectively, which demonstrate an antioxidant activity of 81.2%.

## 1. Introduction

Nanomaterials have many novel applications in biochemistry, biology, engineering, chemistry, medicine, informatics, and electronics, etc. Metallic nanostructures are produced via chemical and physical processes [1]. Among different metal salts and metal NPs, silver nanoparticles (AgNPs) have received tremendous interest in biomedical applications. Furthermore, they are a good disinfectant with no adverse side effects and minimal toxicity in humans [2]. AgNPs are increasingly used in different fields concerning the impacts of silver salts/ions, despite the fact that released Ag ions are vital for antimicrobial activity [3], anti-angiogenesis activities [4], anti-viral activities [5], anti-platelet activities, and antioxidant activities [6]. Furthermore, AgNPs’ antibacterial efficacy is highly dependent on particle size, which could be due to the interaction between the microbial cell surface and NPs, which modifies cell wall and cell membrane characteristics such as permeability and electron transport, resulting in cell death [7,8]. To determine the size, growth, and shape of NPs, capping and stabilizing agents play a significant role in the synthesis of AgNPs.

Various approaches have been used to produce AgNPs, including photochemical and chemical processes in reverse micelles [9], radiation-assisted approaches [10], chemical reduction in a solution of silver salts [11], sonochemical processes [12], electrochemical processes [13], sol-gel synthesis [14], and the microwave heating method [15]. The most common of the synthesis methods mentioned above are chemical methods [16], but these approaches require the introduction of harmful and toxic chemical reducing agents during experiments, which lead to environmental pollution. Plasma reduction is a process in which plasma produces ions, electrons, metastables, charged species, oxidizing species, H_2_O_2_, atomic oxygen, O_3_, OH radical, and UV-radiations, which reduce the metal salts along with stabilizing agents to produce AgNPs of specific sizes [17]. This synthesis technique is eco-friendly and easy to perform compared to other known techniques. Plasma treatment is conducted at atmospheric pressure and room temperature, making it a potentially facile method for the large-scale production of AgNPs.

The plasma reduction process is only suitable for the controlled synthesis of nanostructures when the reaction conditions are carefully set. Several statistical models can be used to optimize the plasma reduction of metal ions into nanoparticles. There has been little literature reported on the statistical optimization of the reaction parameters of the atmospheric pressure DC plasma reduction method for the green synthesis of nanomaterials. In this study, a non-thermal DC plasma reduction technique was used to convert Ag+ ions at the plasma–liquid interface into AgNPs under statistically optimal conditions for biological and photocatalytic applications. By statistically optimizing the reaction parameters using the BBD method, the efficiency and reactivity of AgNPs were improved for different biological [18] and environmental treatment applications. The size of the AgNPs was chosen as a statistical response parameter, while the stabilizer concentration, silver salt concentration, and plasma reaction time were chosen as independent factors. Another major issue in producing NPs using this plasma reduction approach is the selection of a stabilizing agent to passivate or protect the surface of nanoparticles. There are numerous issues that should guide the selection of a stabilizing agent, and these vary significantly from the required morphologies and size ranges of the NPs to the proposed applications [19]. In the reported synthesizing method, glucose was chosen as a stabilizing agent. Glucose is a common and non-toxic ketonic monosaccharide. Due to glucose capping in the synthesis of AgNPs, the resulting AgNPs have high a shelf-life and the uncontrolled growth of nanoparticles is prevented. The stabilized AgNPs have been researched for different high-tech applications, including antimicrobial, antioxidant, and photocatalytic activity [20].

Herein, statistical models were used to investigate and optimize the significant factors affecting the growth of AgNPs and interactions between growth parameters. Statistical models are a viable alternative to traditional one-factor-at-a-time optimization techniques [21]. Both the response surface methodology (RSM) and design of experiments (DOE) methodology have been used to optimize process variables to improve the synthesis process. Although it is employed to investigate the impacts of process variables, these approaches also result in a numerical model that describes the general procedures. Based on its operative effectiveness, the RSM technique is currently widely used to develop various frameworks [13,14,15]. The main novelty of the current study is the optimization of the size of AgNPs by statistically adjusting the process parameters of the plasma discharge reduction technique. In this study, the stabilizing agent, precursor salt, and time of reaction noticeably affected the response variable (the size of AgNPs). The proposed hypothesis of this research was that it is the possible control of the size of AgNPs (their shape and size and the quantitative analysis of the aggregation and agglomeration of AgNPs) due to different process parameters. We also investigated the antioxidant, antibacterial, and photocatalytic activity of the optimized AgNPs.

## 2. Materials and Methods

### 2.1. Materials

AgNO_3_ and glucose (C_6_H_12_O_6_) were supplied by Sigma Aldrich. Pure and deionized water was employed to wash and purify the NPs samples. Glass beakers, a magnetic stirrer and a glass stirrer were utilized to prepare the AgNPs samples. In every examination, 100 mL of the obtained solution was utilized.

### 2.2. Production of Silver Nanoparticles

**Figure 1:**
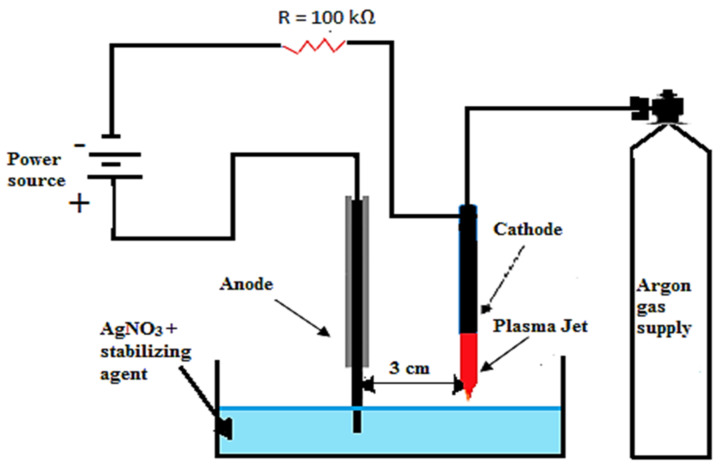
Schematic diagram of plasma synthesized AgNPs.

The plasma discharge setup, employed for the preparation of AgNPs, is illustrated in Figure 1, and is similar to the setup for sucrose-stabilized AgNPs proposed by Altaf and Skiba et al. [22,23]. The reaction solution C_6_H_12_O_6_ (glucose) was used as a stabilizing agent and an aqueous solution of AgNO_3_ (>99.9%, Sigma, St. Louis, MO, USA) as a precursor salt. A silver wire of 6.8 mm diameter was dipped in a 100 mL electrolytic solution, which acted as an anode. A steel needle with a bore diameter of 0.28 mm and a length of 7 cm was used as a cathode over the solution’s surface. Argon gas is used as the major feedstock gas at a fixed flowrate of 200 SCCM. A DC supply was used to spark and sustain the plasma at 4 kV, and the synthesis process was carried out at a fixed current of 15 mA to avoid the melting and overheating effects of the hypodermic needle-tip. For the discharge current, a resistor of 100 kΩ was placed on the ground side of the electrode. The glass beaker and silver anode were rinsed and cleaned by HCL before the experiment. The plasma and solution interaction duration was fixed at various time intervals according to Table 1. The stirring of the solution was avoided, as this has a chance of disturbing the growth rate of NPs. The anode and cathode were fixed at 3 cm apart. The gap turns to conduct and completes the electrical circuit, which initiates the electrochemistry process and the electrolytic solution, prompting AgNO_3_ to dissociate into Ag^+^ and NO_3_^−^ ions. When plasma comes in contact with the solution, if the dissociated solution features Ag^+^ ions, it immediately turns black, which indicates the formation of AgNPs.

### 2.3. Statistical Design for the Synthesis of AgNPs

The statistical optimization of the plasma reduction process for AgNPs was achieved using the response surface methodology (RSM), in which one or more independent variables were involved. RSM provides a greater amount of information with a reduction in the number of experimental trials, chemical doses, time, and total research costs. The interactions between the variables that could impact the production of AgNPs was evaluated using a RSM-based Box–Behnken design [24]. The combined effect of three independently varying variables, namely, the concentration of the stabilizing agent (*A*), the metal salt (AgNO_3_) concentration (*B*) and time of the reaction (*C*), were taken to reveal the statistical analysis for the process at three different levels (−1, 0, +1), which comprised 15 trials. Their findings were fitted into a 2nd order polynomial model. This model was a mathematical relation employed to utilized to obtain the results and approximate the real answer by determining the linear, quadratic, and cross effects of the investigated independent variables, as shown in Equation (1):

(1) $$Y\;=\;\beta_{0}\;+\;\beta_{1}A\;+\;\beta_{2}B\;+\;\beta_{3}C\;+\;\beta_{12}AB\;+\;\beta_{13}AC\;+\;\beta_{23}BC\;+\;\beta_{11}A^{2}\;+\;\beta_{22}B^{2}\;+\;\beta_{33}C^{2}$$

Here, *Y* denotes the outcome variable, which is the size of AgNPs, and *β_0_* is a regression coefficient at the central point. Similarly, linear coefficients are denoted by *β_1_*, *β_2_*, and *β_3_*, 2nd order interaction coefficients are denoted by *β_12_*, *β_13_*, and *β_23_*, and quadratic coefficients are denoted by *β_11_*, *β_22_*, and *β_33_*. The independent variables are labeled as *A*, *B*, and *C*. The effect of independent variables was quantified on the outcome variable/response parameters, which was the size of nanoparticles. The levels of individual variables were decided based on primary results produced during experimentation, including optimization via three-factor design. The composition of the trials and the findings of the performed experimental work obtained by applying a Box–Behnken design matrix is shown in Table 1. Statistical analysis of the obtained results was performed using Minitab Software.

### 2.4. Characterization of AgNPs

Under optimal reaction parameters, the physiochemical properties of the synthesized AgNPs were evaluated. The most suitable combination of molarity and synthesis time for the glucose-capped AgNPs was preferred. The obtained product was annealed at 60 °C in a vacuumized oven to obtain fine powdered AgNPs. The crystallinity and size of as-synthesized NPs were investigated via XRD at 40 kV and 150 mA. The intensity of Cu *α* radiation (0.15406 nm) was selected and a diffractogram (X’Pert Pro-PANlytical) was obtained in the 2*θ* range of 20 and 80°. UV–Vis spectrophotometry (UV-1800 Parma Spec., Shimadzu) was employed to study the optical characteristics of the optimized AgNPs in the absorbance range of 400–800 nm. The measurements were carried out at room temperature for different reaction times. After mixing the precursor salt and stabilizing agent in a prefixed set of volumes, the color variation from transparent to black was a confirmatory indication of AgNP formation. Different reaction solutions were obtained under different molar concentrations of AgNO_3_, stabilizing agent concentration, and reaction time of with the help of statistical design. SEM analysis was used to examine the superficial characteristics of the AgNPs using (JSM 6380A, JEOL Ltd., Tokyo, Japan). The elemental composition of the nanoparticles was examined with EDX apparatus (JOEL, JSM-6480A) operated at a 20 kV. The optimized (small-sized) AgNPs were subjected to FT-IR (JASCO FTIR, Tokyo, Japan) analyses to study the adsorption of functional groups onto the surface of the AgNPs. The obtained nanoparticles were separated from the solution through centrifugation using a HERMLE-Z323K machine and were then suspended in deionized water. FTIR analysis was performed using KBr pellets for the purpose of characterizing the optimized AgNPs. IR absorption analysis was performed from 4000–500 cm^−1^.

### 2.5. Antimicrobial and Antioxidant Activity

A disc diffusion test was performed to measure an antibacterial assay for AgNPs produced under optimized conditions. The antibacterial activity was measured by using *E. coli* (Gram-negative) and *S. aureus* (Gram-positive) strains. In total, 10 μL of AgNPs (runs 1, 2, 4, and 12) in a concentration of 150 μg/mL were loaded in each disc. An analysis of the inhibition zone was carried out at 37 °C for 24 h of incubation. As a result, the zone of inhibition was calculated in millimeters.

The AgNPs of statistical runs 1, 2, 4, and 12 showed a good ability to scavenge stable radicals. The antioxidant activity of these nanoparticles was tested using the 1,1-diphenyl-2-picrylhydrazyl (DPPH) radical. Using the stable radical DPPH, the free radical scavenging activity of AgNPs and conventional vitamin C was investigated. A total of 1 mL of varied AgNPs solutions (runs 1, 2, 4, and 12) were vortexed extensively with 1 mL of newly made DPPH (1 mM in methanol) solution. This was then incubated for half an hour before an absorbance test was performed a fixed wavelength of 517 nm. The results were expressed in the form of the percentage inhibition of the DPPH radical. The control was DPPH (all reagents except the sample) and the blank solution was methanol. The inhibition percentage (% *DPPH*) was obtained to express the free radical scavenging activity using Equation (2) [25]:

(2) $$\%\;DPPH\;=\;\frac{O_{c}\;-\;O_{s}}{O_{c}}\;\times \;100$$

where *O_c_* and *O_s_* are the absorbance of control and AgNPs/vitamin C, respectively.

### 2.6. Photocatalytic Degradation

The optimized AgNPs (run 4) was used as a catalyst for the catalytic removal of methyl orange (MO) under visible light exposure [22]. MO stock solution was made by dissolving 50 parts per million of dye in 100 mL of distilled water. Then, 5 ppm solution of methyl orange was taken from the stock solution and 0.1 mL of hydrogen peroxide and 0.1 g dose of catalyst were added to the solution to promote the degradation of dye. After this, the aqueous solution was agitated for half an hour in a dark environment to complete the adsorption–desorption equilibria. The experiment was performed under a sunlight irradiation of 100 min. Then the solution was poured into a cuvette, the spectra were scanned in a 200 to 800 nm wavelength range, and photometry was performed to check the MO reduction process at a 400 nm wavelength.

## 3. Results and Discussions

### 3.1. Optimization of Size of AgNPs through RSM Analysis

**Figure 2:**
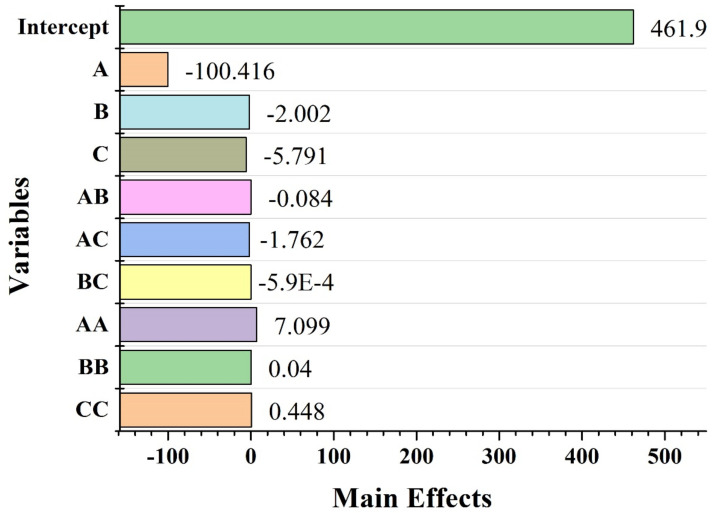
Main effects plot graph for the size of AgNPs.

**Figure 3:**
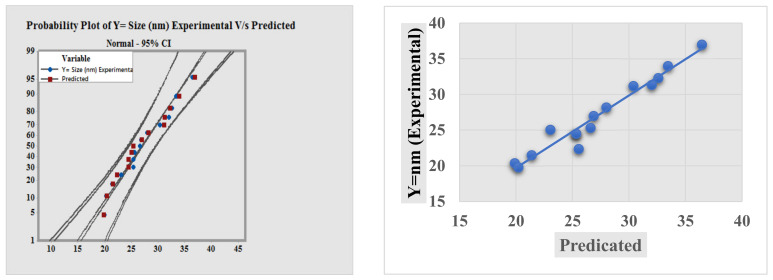
Normal and probability plot for experimental vs. predicted size of AgNPs.

The BBD approach was used to determine the possible average size of metal nanoparticles (MNPs) affected by selected process parameters, including concentration of stabilizing agent, metal salt concentration, and time of the reaction mixture. The RSM approach was adopted for the optimization of an average size for the prepared AgNPs calculated by the regression equation. Overall, 15 sets of trials were created by BBD (Table 1) using three process parameters at three different levels. To examine the effect of these parameters, the experimental results were statistically analyzed using ANOVA (analysis of variance). The results of ANOVA analysis are shown in Figure 2 in the form of a Pareto chart. Each bar length in the Pareto chart indicates the significant influence of a respective factor and its effects on the considered response (*Y* = size of AgNPs), as indicated by F-values in Table 2. The model F-value (16.039) in Figure 2 confirms the significance of the ANOVA analysis model. The parameters with a *p*-value less than 0.05 were observed to impact the response substantially. The model was deemed statistically valid because the *p*-value did not exceed 0.002. The adjusted-R^2^ value (0.7750) was also significant, indicating the validity of the adopted model. But the resulting R^2^ value (0.9016) revealed the 90.16% contribution of independent variables in the total variation in the average diameter of NPs [26]. In terms of statistic design, our objectives were to identify the maximum significant interactions between process parameters to generate small-sized AgNPs of optimum uniformity and stability. It was based on the analysis of the normal residual graphs for each of the response variables shown in Figure 3.The fitness of the model was checked by calculating the coefficient of determination (R^2^). A correlation coefficient R^2^ ~ 1 value indicates a strong synergistic effect [17]. The observed and predicted R^2^-values of 0.96646 show that the response has (96.64%) variability. As a result, the current R^2^ value shows that the predicted and actual responses are aligned well, implying that the model is dependable in the present research. Multiple regression analysis was used to determine all of the model coefficient values of the quadratic model. The *p*-value and Student *t*-test suggested a satisfactory quality in terms of statistical analysis (Table 3). The linear impacts of reaction salt concentration (*B*) and reaction time (*C*) showed a negative impact on the size of AgNPs, whereas the stabilizing agent concentration (*A*) revealed a positive impact. The importance of each of the coefficients was assessed by the *p*-value, which was found to be significant in the context of understanding the pattern of reciprocal interaction between process variables. The lesser the *p*-value, the more significant the correlated coefficient. Among all the linear effects, the linear term of AgNO_3_ (*A*) had the most significant negative influence on nanoparticle size according to the degree of significance. The quadratic terms for AgNO_3_ (*A^2^*), the concentration of the stabilizing agent (*B^2^*), and reaction time (*C^2^*), on the other hand, all showed a considerable positive influence, indicating that they may be treated as limiting factors, with slight fluctuations in their values affecting the size of AgNPs. In line with this, the Pareto charts revealed that only the interaction between the stabilizing agent concentration (*A*) and reaction time (*C*) had a significant negative effect. To forecast the particle size in the experimental runs, a 2nd order polynomial model was fitted to the results of the experiments using the Minitab software. This model develops an empirical link between the response variable and the three independent factors, as in Equation (3):

(3) $$Y_{\left(AgNPs\;size\right)}\;=\;230.981\;-\;50.208A\;-\;1.00097B+2.895C\;-\;0.042AB\;-\;0.79AC\;-\;0.00029BC\;+\;3.549A^{2}\;+\;0.0201B^{2}\;\;+\;0.224C^{2}$$

The sign of the coefficients determines the response performance. In this scenario, a decrease in particle size revealed positive coefficient values, while a negative coefficient showed an increase in particle size. Furthermore, the effect of a variable is more significant when the absolute value of a coefficient is high. As a result, according to the preceding equation, the concentration of the stabilizing agent significantly impacted the particle size. All possible combinations of input variables achieved by BBD are able to optimize the response surface curves that are plotted by the Minitab software. The ANOVA analysis confirmed the high significance of the quadratic model, with a *p*-value of lesser than 0.05. Since some of the *p*-values were found to be insignificant, the model was reduced using the RSM methodology. The obtained results are summarized in Table 2.

The impacts of process factors on the response variable were determined using 3-D surface plots. By visualizing the interaction of two test factors with a constant third variable, these 3-D surface plots helped in determining the optimized values of process variables for the production of AgNPs through the plasma reduction method. The relevance of the plots between the selected variables is reflected in the shape of the 3D plots, which can be circular, round, or saddled [27].

**Figure 4:**
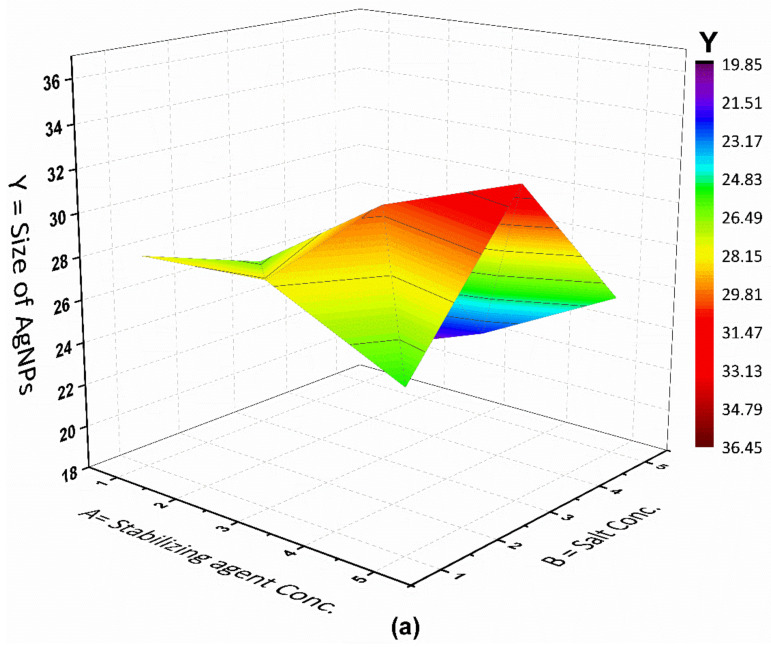

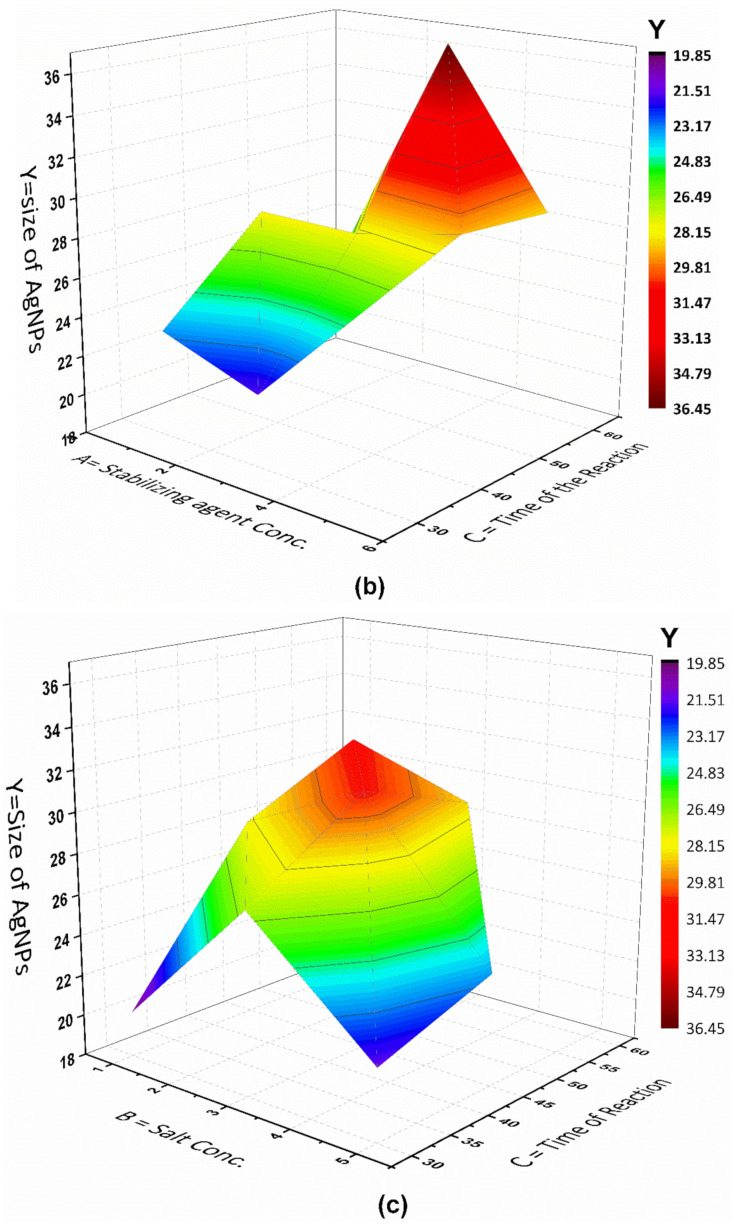
(**a**–**c**): 3D surface plot showing the effect of process parameters on the size of AgNPs.

Figure 4 depicts a 3D surface plot of the variables for the purpose of investigate the role of independent variables in shaping the particle size (response variable). Graphs were constructed in this context by displaying the response versus effective components to show binary interactions. The remaining parameters remained unchanged at the central level of the BBD. Figure 4a depicts the interaction between stabilizing agent (*A*) and metal salt (*B*) concentrations, revealing that the average diameter size reduced as the metal salt and the stabilizing agent concentrations increased. This could be explained by increasing the concentration of metal salts to a specific point, allowing for faster particle production and smaller NPs. Furthermore, the particle size grew as the metal salt concentration rose. This could be due to an increase in the agglomeration of growing nanoparticles [28]. Khan et al. reported the nucleation synthesis of NPs of a specific size with both a reduction in and stabilization of chitosan. The study revealed the production of smaller-sized NPs by increasing the concentration of Ag^+^ ions and stabilizing agent [29].

The middle levels of both the stabilizing agent concentration and the metal salt concentration (3 mM, 5 mM) were employed to produce the smaller-sized particles. The size of the NPs gradually increased as the number of ions increased. Increased particle sizes and a loss of stability are caused by denaturation, inactivity, poor reductive enzyme activity, and other reactive molecules involved in the synthesis process, or by an inappropriate stabilizing agent concentration [30,31]. The synthesis process, carried out using the highest concentration of independent variables, produced relatively larger nanoparticles. The higher the concentrations of the stabilizing agent and the metal salt, the greater the likelihood of generating large-sized AgNPs due to the ability of the stabilizing agent to deliver electrons to the oxidizing agent and reduce the silver ions into nanoparticles [29]. Similarly, the size of the optimized nanoparticles was generated using AgNO_3_ [5 mM], stabilizing agent [3 mM], and a reaction duration of 30 min. This is because the synthesis process influences the role of reagents in determining the size and stability of nanoparticles, which differ depending on the methods utilized to inhibit their aggregation. Furthermore, the size of the nanoparticles was discovered to be dependent on the nucleation rate and the growth of nanoparticles, which can be influenced by variables such as the stabilizing agent. The mutual interaction between the stabilizing agent concentration (A) and the reaction time (C) on AgNPs synthesis is depicted in Figure 4b. The average particle size reduced as the stabilizing agent concentration increased. However, the size increased slightly as the reaction time increased. This could be because saccharide-coated AgNPs are inactive [32]. In general, time indicates the continued reduction of silver ions and increase in the concentration of AgNPs [33]. Restrepo et al. generated tiny, monodispersed metal nanoparticles by using the least amount of stabilizing agent required. The nature and amount of the stabilizing agent added, which can influence the equilibrium present on the particle surface, can therefore be correlated with the size, morphological, and electrochemical features of the NPs, i.e., the type and amount of stabilizers do not modify the reaction mechanism but affect the dispersion and can modify the textural characteristics of the metallic NPs [34]. According to the results, run 4, with a high concentration of stabilizing agent and a reaction period of 30 min, produces the smallest sized AgNPs. Another study by Smiechowicz et al. investigated the chemical approach to optimize the process variables and generate a high yield of AgNPs. The following settings were used to obtain the highest yield of AgNPs: the starting pH of the solution was 7; the amount of reducing agent was 1%; the concentration of AgNO_3_ was 1 mM; the reaction time was 3.5 min; and the stirring time was 15 min. The AgNPs obtained had excellent antibacterial activity [35]. Figure 4c shows the effect of metal salt conc. (B) and reaction time (C) on the response variable (the size of AgNPs). It showed that an increase in the concentration of metal salt and reaction time supported the production of lower-sized AgNPs. Using average reaction time and metal salt concentration values produced the smallest size (30 min, 5 mM). The increased nucleation process rate caused by a significant quantity of OH^−^ could be attributed to the production of tiny AgNPs. The results of this work agree with the findings of Wypij et al. [36]. They revealed that the production of AgNPs and their physiochemical properties are affected by reactant concentration, temperature, pH, and reaction duration. It was discovered that modifying these parameters, which control the nucleation rate, growth of nanoparticles and precursor, can change the size and nature of the produced nanoparticles. The current investigation of the influence of the direct connection between these parameters on the response represents the effect of the stabilizing agent concentration, metal salt concentration, and reaction duration on the production of AgNPs. Solving the regression equation in the Minitab software revealed smaller AgNPs under optimum conditions. The ideal values for the variables were a 30 min reaction time, 3 mM of stabilizing agent, and 5 mM of metal salt precursor. The smallest AgNPs size according to the model was 19.89 nm, which is achieved by optimum conditions provided above.

### 3.2. XRD Analysis

**Figure 5:**
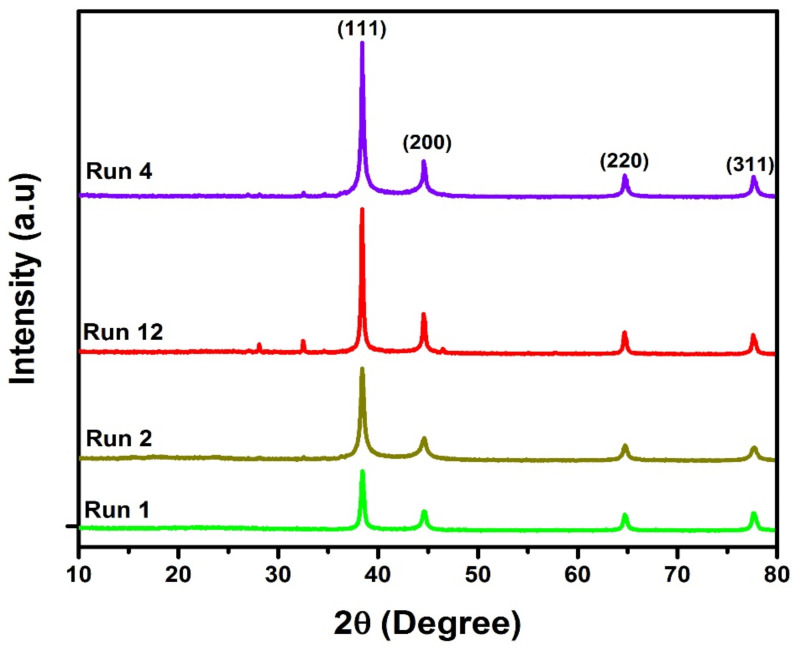
XRD spectra of statistical analyzed AgNPs (runs 1, 2, 12 and 4).

The formation and purity of metal silver are confirmed by powder X-ray diffraction (XRD) analysis. To elucidate the structure of AgNPs, the XRD analysis of AgNPs synthesized under optimum conditions was performed. Figure 5 shows the XRD patterns of optimized samples with 2-theta values in the range of 20° to 80°, prepared by the plasma reduction process. The XRD peaks showed FCC planes (111), (200), (220), (311), and (222) corresponding to the diffraction peaks at 2θ of 38.39°, 44.63°, 64.71°, and 77.59°, respectively. These planes confirmed the crystalline character of the synthesized nanoparticles. No secondary phases or peaks except for AgNP peaks were observed in the XRD patterns. This indicates the formation of pure AgNPs without any impurity phase. These findings agree with the published literature on the plasma synthesis of AgNPs [17,37]. The average size of plasma-reduced AgNPs was calculated by Debye–Scherrer’s Equation (4):

(4) $$D\;=\;\frac{\lambda K}{W\;Cos\theta }$$

where *D* is the average crystallite size, *W* is the full width at half maximum (FWHM), θ is the diffraction angle, and *λ* is the X-ray wavelength (0.1541 nm).

The peaks with strong intensities confirm that the particles had a good level of crystallinity. The highly intense diffraction peak, computed at 2*θ* = 38.39, corresponds to the (111) plane, which is used to determine the average size of the AgNPs, which was approximately 19.89 nm. The XRD results indicated that the AgNPs were highly crystalline, with a FCC crystal structure that was found to be consistent with previously published findings [38,39].

### 3.3. UV–Vis Analysis

UV–Vis spectroscopy is primarily an optical analysis technique and was used in this study to investigate the optical characteristics of plasma-reduced AgNPs.

**Figure 6:**
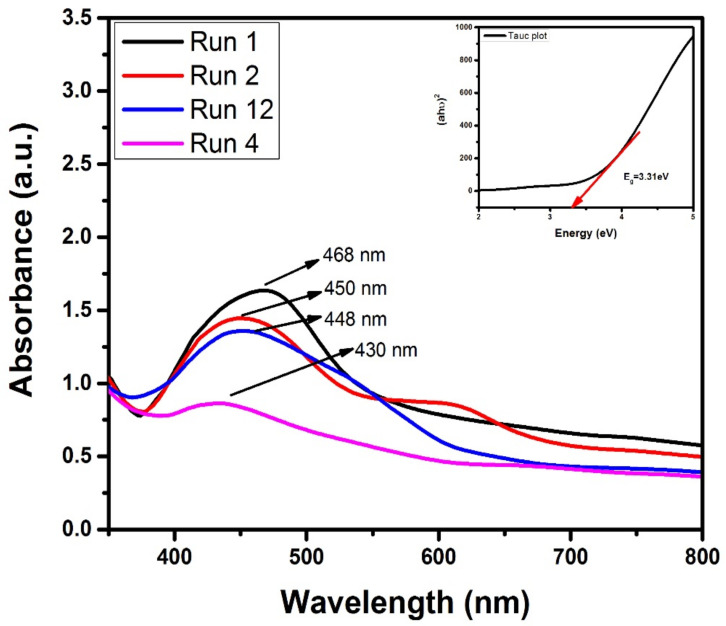
Variation in the UV-absorption peak with different process parameters.

Since the valence and conduction bands of AgNPs are close to each other, electrons can easily move between them. Figure 6 shows the absorption spectra of AgNPs with different process parameters (mentioned in Table 1). The particle size and physio-chemical characteristics have a significant impact on the absorption spectra. The sample exhibited surface plasmon resonance (SPR) characteristics. AgNPs with particle sizes ranging from 5 to 25 nm displayed a narrow band, with the highest absorbance, 468 nm. Thus, the absorption spectra were found to be strongly dependent on dipole oscillation [40]. The literature reveals that the SPR peak shifts to a shorter wavelength when the size decreases. It has also been observed that when the sample size decreases, the absorption spectra weaken and become broader [41]. This plasma reduction process results in the optimized production of AgNPs with a diameter of 19.89 nanometers. Furthermore, the absorption spectra of the AgNP samples denoted by runs 1, 2, 12, and 4 in Figure 6 show a maximum absorption peak at 468 nm.

The statistically combined effect of the three active input variables, namely the concentration of stabilizing agent (A), the metal salt concentration (B) and the reaction time (C), reveals the high intensity of the absorption peak in the absence of the stabilizing agent. The absorption intensity decreased in the presence of the stabilizing agent. The smaller UV absorption peak, of approximately 600 nm, in run 2 might be due to the presence of some traces of stabilizing agent in the sample. Such traces can be removed by washing the nanoparticles multiple times with distilled water. Another possible reason for the appearance of two peaks in the UV spectrum is the transition of electrons due to multiple energetic excitations from the light source.

Different concentrations of AgNO_3_ result in the production of non-uniformly distributed NPs of different sizes (Table 1). The shifting of the absorption edge from 430 to 468 nm indicates the production of large NPs. On the other hand, as the concentration of AgNO_3_ increased from 1 mM to 5 mM, the absorption edge shifted toward a lower wavelength, indicating the production of small AgNPs. An absorption peak was observed in the visible range (430 nm) of the electromagnetic spectrum, which is in good agreement with the results reported by the other researchers [42]. Without using any further information, the optical band gap of plasma-reduced samples is obtained by fitting the absorption spectra to the reference absorption spectra [43]. The band gap energy of optimized AgNPs is determined by the Tauc plot Equation (5):

(5) $${\left(ah\upsilon \right)}^{2}\;=\;A\left(h\upsilon \;-\;E_{g}\right)$$

where *E_g_* is the band gap energy and *λ* is the wavelength. The band gap value of plasma-reduced samples was measured to be approximately 3.31 eV using the Tauc relation and is shown in Figure 6.

### 3.4. SEM and EDX Analysis

**Figure 7:**
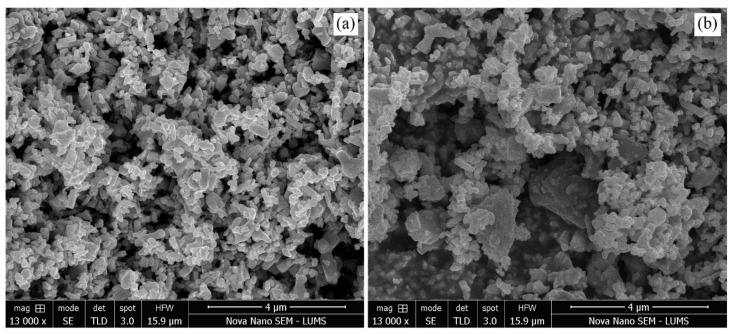
(**a**) SEM micrograph of run 4 (small-sized AgNPs), (**b**) SEM image of run 6 (large-sized AgNPs).

**Figure 8:**
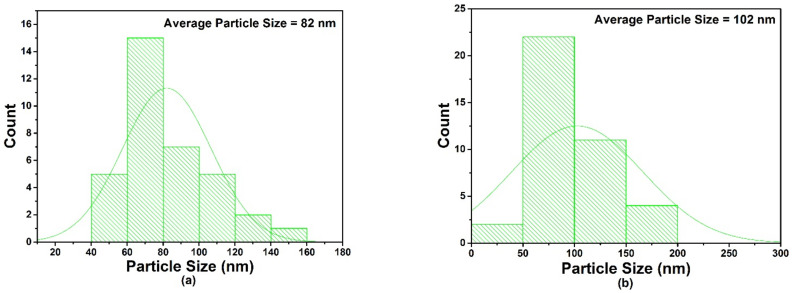
(**a**,**b**) Size distribution histogram of AgNPs.

**Figure 9:**
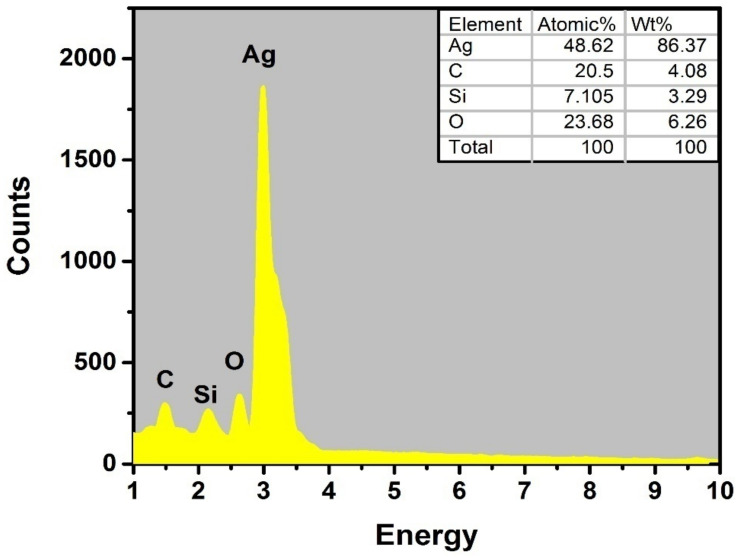
EDX spectra of optimized AgNPs (run 4).

SEM analysis was used to investigate the sizes optimized AgNPs under different combinations of process parameters. Figure 7a,b shows the SEM micrographs of plasma reduced, size-optimized AgNPs. Smaller and spherical-shaped AgNPs with an average size of 19.89 nm were obtained under smaller-sized parameters, as shown in Figure 7a. On the other hand, irregular-shaped and slightly larger-sized agglomerates of AgNPs were obtained with the constant concentrations of both of the parameters (3 mM-glucose/3 mM AgNO_3_), with an average AgNP size of 33.3 nm, as shown in Figure 7b. The selected process parameters did not significantly impact morphological features; small agglomerates were obtained, but the spherical shape was dominant overall. It may be suggested that the prepared AgNPs were adequately separated from each other, with spherical shapes, and were free from aggregation. Thus, under optimized conditions, the current plasma reduction approach was used to prepare AgNPs using glucose as a stabilizing agent [26]. The mean value and size distribution of the AgNPs were then calculated and plotted via the image-processing technique in the form of histograms, as shown in Figure 8. The mean particle diameter of the AgNPs was calculated from the SEM analysis, the image-processing technique used in this study, and then compared with the mean particle size attained by XRD analysis. The particle size results can be found in Table 4. Figure 9 shows the elemental compositions of the size-optimized AgNPs (run 4) analyzed by energy dispersive X-ray spectroscopy (EDX). The detected elements from the EDX spectrum were: Ag, which confirms the formation of AgNPs, carbon peaks (which may be due to the glucose used as a stabilizing agent or use of a carbon film during EDX measurements), and silicon and oxygen (due to the silicon dioxide (SiO_2_) wafer on which the glucose-AgNPs were deposited). The detected elemental composition also confirmed the FTIR results. Oxygen signals originate from glucose and AgNO_3_, which are used as stabilizing agents or precursors as salt in the synthesis of AgNPs. The elemental profile showing a significant peak at 3 keV confirms the formation of pure AgNPs along with the minor impurity content of the stabilizer.

### 3.5. FTIR Analysis

**Figure 10:**
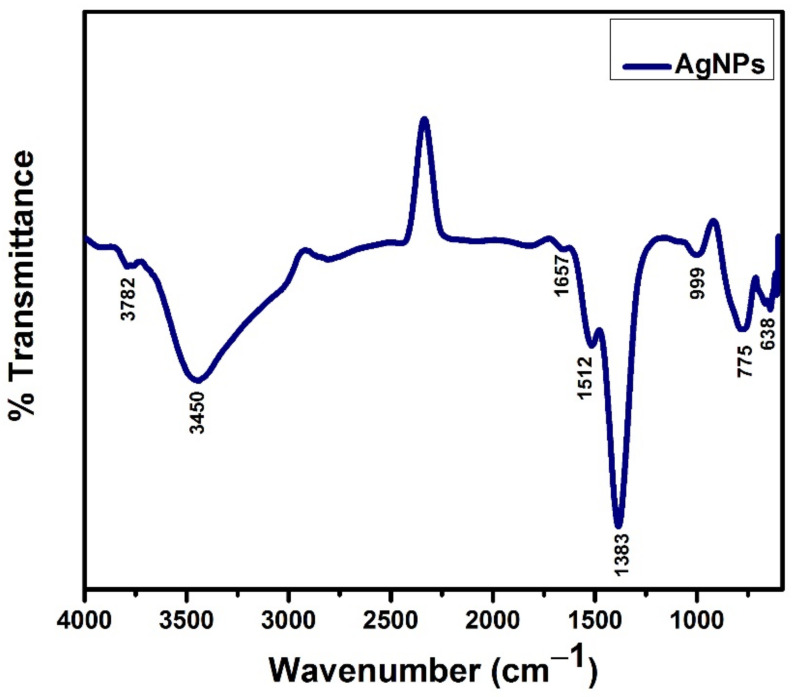
FTIR spectrum of optimized (run 4) AgNPs.

The FTIR spectroscopy of optimized AgNPs (run 4) was used to analyze their purity and associated functional groups, as illustrated in Figure 10. The FTIR of AgNPs indicates eight representative peaks at 638 cm^−1^, 775 cm^−1^, 999 cm^−1^, 1383 cm^−1^, 1512 cm^−1^, 1657 cm^−1^, 3450 cm^−1^, and 3782 cm^−1^. It has been observed that noble metal ions (Ag^+^) speed up the oxidation process and reduce themselves into silver atoms. The natural sugar in glucose also serves as a surface coating for synthesized NPs. A glucose stabilizer, which is also considered a small molecule, is an eco-friendly and porous stabilizer that allows small molecules to be diffused. When it is attached to the surface of nanoparticles, it is easily removed during the plasma reduction process. As a result, the plasma reduction process produces NPs that are more biocompatible and purer. During the synthesis process, silver ions may interact with the binding sites of bioactive chemicals existing in the glucose. The shift in the position of a peak at the 1383 cm^−1^ band indicates the presence of the S–O group.

The slight stretching at the 1657 cm^−1^ peak can be attributed to the symmetric and asymmetric stretching of the -COO- group. This indicates the presence of the infinitesimal amount of glucose stabilizer. The band stretching at 1512 cm^−1^ can be ascribed to the -OH bonding of the acid group. The absorption band stretching at 3450 cm^−1^ represents the presence of the OH functional group. The band stretching near 999 cm^−1^ indicates the presence of alcohols in the sample. The band stretching at 775 cm^−1^ represents out-of-plane stretching in C-H aromatics. The band stretching at 638 cm^−1^ can be ascribed to the C-C bond of alkenes. The contribution of glucose utilized in the stabilization process results in the production of pure AgNPs and slightly shifts the peak positions of the -COO- and -OH groups at 1657 cm^−1^ and 3450 cm^−1^, respectively.

### 3.6. Antibacterial

**Figure 11:**
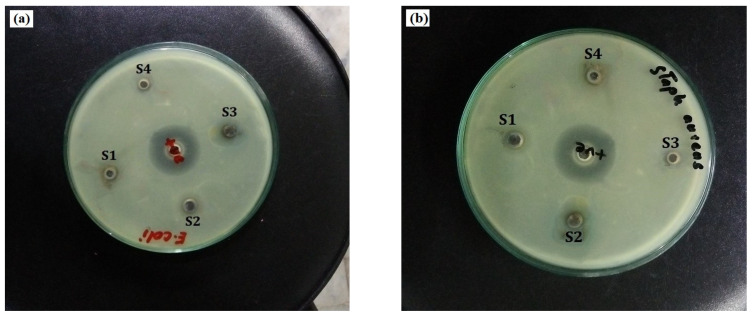
(**a**,**b**) The images represent the antibacterial activity of optimized AgNPs against Gram +ive and Gram −ive bacterial strains.

The antibacterial mechanism of AgNPs involves the release of silver ions from the nanoparticles, which contribute to the bactericidal effect. Silver ions, which are typically utilized in the form of nanowires, nanolayers, and nanoparticles, are well known for their antibacterial and antimicrobial activities [44]. The antibacterial activity of AgNPs was assessed by measuring the diameter of the generated clear zone and the width of the inhibitory zones in mm. *E. coli* strain showed a relatively smaller antimicrobial zone of 12.5 mm while *S. aureus* showed a larger antibacterial zone of 18.9 mm. The difference in the inhabitation zones of these bacteria can be attributed to the difference in the compositions of their cell walls. Figure 11a,b shows the influences of the selected runs, 1, 2, 4, and 12, on the bactericidal property of the resulting AgNPs against *E. coli* and *S. aureus*.

The optimized AgNPs with a size of 19.89 nm showed the best antimicrobial activity against *E. coli* and *S. aureus* bacteria. Because of their vast reactive surface, smaller nanoparticles had a better antibacterial effect than larger-sized nanoparticles. Identical results were obtained for antimicrobial activity. NaBH_4_ + PVP K-30 silver nanoparticles showed a larger zone of inhibition compared to other systems due to their smaller size, as reported by Agnihotri et al. [45]. Moreover, previous researchers reported how the size and shape of nano-sized entities influence the antibacterial activity of AgNPs. Dong et al. [46] and Pal et al. [47] reported that triangular-shaped AgNPs had the strongest antibacterial activity when compared to spherical ones. They reported the stronger reactivity of triangular AgNPs, which was attributed to their geometrical structure and (111) crystal planes. The maximal antibacterial productivity is achieved by high-atomic-density (111) facets. Gunawan et al. studied the immobilization of AgNP/CNTs coated on the surface of a hollow fiber membrane made of polyacrylonitrile (PAN). PAN alone and AgNP/CNT/PAN membranes were tested for the filtration of feedwater contaminated with *E. coli*. The AgNP/CNT coating considerably improved the antibacterial activity and antifouling capabilities of the membrane [48]. Silver nanoparticles synthesized from *T. harzianum* culture filtrate were stable and had beneficial bioactivities. Green-synthesized AgNPs were found to have wide antibacterial action against both Gram-positive and Gram-negative pathogenic bacteria, as reported by Konappa et al. [49].

A positively charged silver ion may form an electrostatic attraction with a negatively charged microbial membrane. Table 5 shows the zone inhibition results for samples S1 (run 1), S2 (run 2), S3 (run 12), and S4 (run 4). The inhibition zones of AgNPs for different metal salt concentrations were measured to be approximately 5 mm, 6 mm, 9 mm, and 12 mm when the *E. coli* strain was used. This was also expressed as AgNP size < followed by a zone of inhibition. Similarly, the inhibition zones of AgNPs against *S. aureus* were measured to be approximately 8 mm, 10 mm, 12 mm, and 18 mm, with varying concentrations of stabilizing agents and metal salts. Figure 11a,b indicate the bactericidal effect of the resulting AgNPs towards *S. aureus* and *E. coli* depending on the different process variables. It would appear that at a 5 mM metal salt concentration and 3 mM of stabilizing agent, the number of formed nanoparticles was greater and the agglomeration of the formed AgNPs was limited due to the concentration of the metal salt and the high concentration of natural stabilizers such as saccharides, carbohydrates, and proteins that were present in the synthesis of the AgNPs. As a result of this condition, optimized NPs exhibit smaller particle sizes and higher surface area to volume ratios, which results in more of the produced NPs becoming attached to the cell membrane, induced cell death, and an altered permeability. Smaller-sized NPs can easily infiltrate microorganisms and disrupt their function by attaching to nucleic acid and linking to the functional areas of specific enzymes [50].

**Figure 12:**
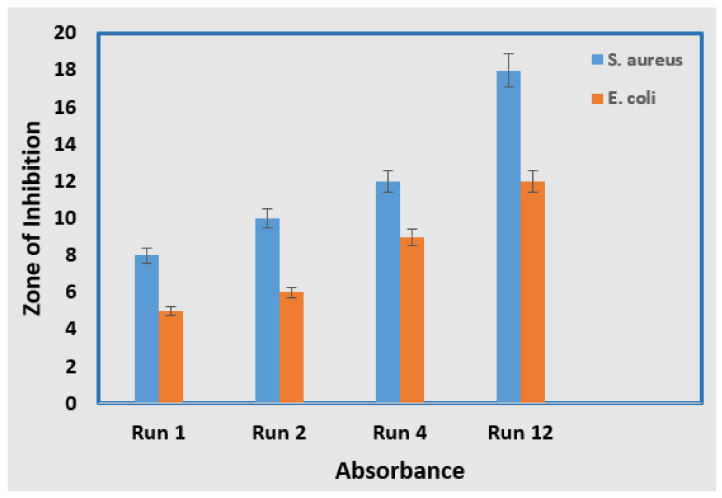
Graph shows the maximum zone of inhibition against the *E. coli* bacterial strains.

The strong antibacterial effect of glucose-stabilized AgNPs against *S. aureus* is slightly stronger than their effect against *E. coli* due to a greater zone of inhibition, as indicated in Figure 12. The effective zone of different samples was measured to be approximately 18 mm and 12 mm against both bacterial colonies, respectively. This demonstrates that the antibacterial activity of AgNPs is dependent on particle size when the stabilizing agent and metal salt concentrations were used with various concentrations. The obtained results agree with the findings of Skiba et al. [51]. They illustrated that synthesized AgNPs possess an improved antibacterial effect against Gram-positive and negative bacteria, which is consistent with previous reports [18,52,53].

### 3.7. Antioxidant

**Figure 13:**
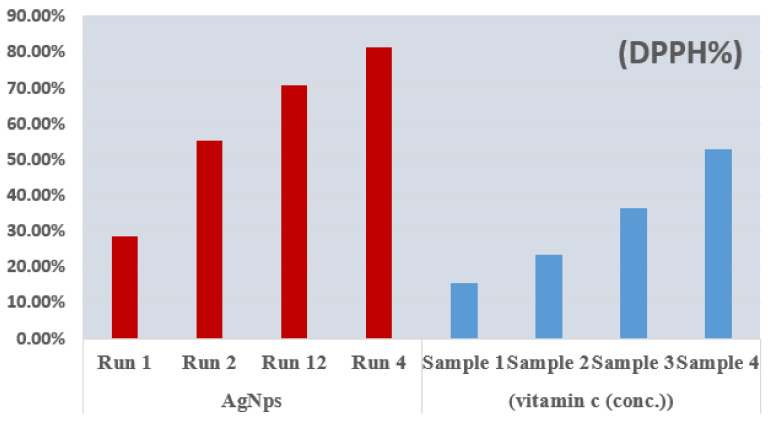
DPPH scavenging activity of optimized AgNPs, with different concentrations of vitamin C for comparison.

The antioxidant activity of optimized AgNPs was conducted to compare them with standard vitamin C. The previously reported protocol was adopted with a number of modification to perform the antioxidant assay [54,55]. It is possible to obtain smaller AgNPs using optimum values for selected variables. The ideal values for the variables were 30 min for the reaction, 3 mM for the stabilizing agent, and 5 mM for the metal salt. Figure 13 showed the antioxidant activity of the optimized AgNPs for runs 1, 2, 12, and 4 compared with vitamin C.

As presented in Figure 13, with lower amounts of stabilizing agents and decreased amounts of AgNO_3_ salt, the antioxidant activity of the synthesized NPs did not increase. However, at fixed and higher concentrations of glucose, the antioxidant activity of the plasma-reduced AgNPs increased with rising metal-salt concentrations. The obtained results can be explained by the fact that, at high concentrations of metal-salt (i.e., the concentration of the main organic compound, for example, as glucose), the inhibition was found to be higher in the case of the plasma-reduced AgNPs when compared with vitamin C. Thus, the current study indicates that by increasing the metal-salt concentration, the antioxidant activity of glucose stabilized AgNPs can be improved. Similar observations were noted for the antioxidant activity of small-sized AgNPs, which were in good agreement with reported studies [25,56]. There have been a small number studies focused on the antioxidant activity of AgNPs concerning the size, shape, and structure of the material. For example, Konappa et al. reported significant antioxidant activity in the case of green-synthesized AgNPs using DPPH free radical scavenging and FRAP assay [49]. Kandiah et al. reported the green synthesis of AgNPs from all six varieties of *Catharanthus roseus* at room temperature for 3 days. They investigated antioxidant activity using the TFC, TPC, TAC, DPPH, FRAP, and IC50 assays. Their findings showed higher antioxidant activity with respect to the AgNPs compared their respective water extracts [57]. The antioxidant activities AgNPs and AgMPs using maleic acid and citric acid as capping agents were evaluated by 2,2-diphenyl-1-picrylhydrazyl (DPPH) assay. The findings revealed that the modifiers have an effect on the size and shape of AgNPs and, consequently, their antioxidant and antibacterial activities [58].

### 3.8. Photodegradation of Methyl Orange (MO) Dye with AgNPs

**Figure 14:**
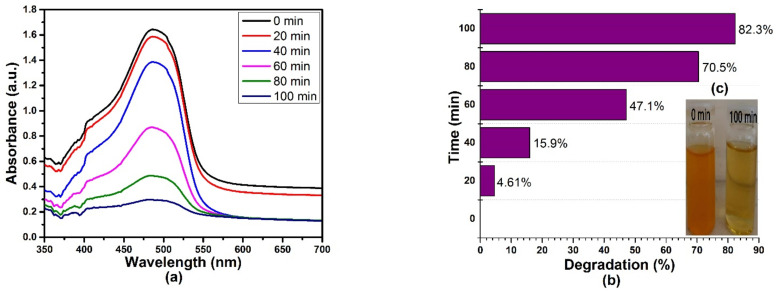
(**a**) Absorbance spectra of an aqueous solution of MO dye treated with (run 4) optimized AgNPs at 100 min of irradiation, (**b**)% of dye degradation at different time intervals, (**c**) image of 0 min and 100 min degradation of MO.

The photocatalytic activity of the size-optimized AgNPs (run 4) was tested by the degradation of methyl orange (MO) solution under sunlight irradiation. Furthermore, the degradation of the dye was monitored by a UV–Vis Spectrophotometer. As can be seen in Figure 14a, in the presence of 0.1 g of an AgNP photocatalyst, the intensity of the peaks at 465 nm gradually decreases and disappears within 100 min for 5 ppm of MO dye. Figure 14b,c show that 82.3% degradation of MO was achieved under sunlight illumination, and the dye degradation was identified by a change in color in an aqueous solution. The results revealed that the optimized AgNPs effectively degraded MO dye (82.3%) after 100 min of degradation. The dye degradation efficiency of the AgNPs increased with an increase in time intervals and was calculated according to the following formula in Equation (6) [59,60]:


(6) $$\%\eta \;=\;\frac{A_{0}\;-\;A_{t}}{A_{0}}\;\times \;100$$

In this formula, *A_0_* and *A_t_* denote the absorbance of the dye samples collected at 0 min and after a specific time period t (min), respectively. Solar irradiation is more effective than other irradiation sources for the decomposition of MO in the presence of nanosized metal catalysts [59,61].

According to the photocatalysis mechanism, visible light approaches the valence electrons of AgNPs. They receive energy and get emitted from the valence shell. These emitted electrons are highly energetic electrons (O-atoms) that generate hydroxyl radicals, leaving the Ag^+^ ions dragging towards the anionic MO dye. This results in the absorption of generated hydroxyl radicals onto the surface of the AgNPs, thereby oxidizing MO atoms into their degraded constituents. Hashemi et al. reported the degradation of MO by adding different amounts of AgNPs@SEE (10 and 15 µL) photocatalyst under sunlight irradiation. Their results showed that the increase in the degradation of MO was more significant with an increase in the irradiation time compared to the effect of increasing the concentration of the catalyst [62]. Another study by Lin et al. involved the fabrication of graphene–Ag nanoparticle-based hybrid structures for novel electro-optical devices. Their findings showed that heated holes on graphene play a primary role in plasmon–exciton co-driven oxidation processes. The importance of hot electrons in oxidation reactions is minor. Plasmon–exciton coupling enables co-driven surface catalytic processes, in which the gate voltage controls the density of states of holes and electrons on graphene, while the bias voltage drives the kinetic energy of holes and electrons or current [63]. Table 6 provides a summary of AgNPs used in different applications.

**Table 6:** Summary of AgNPs-based applications depending on their properties.

Sr No	Material	Method	Size/Shape/Structure	Antibacterial	Antioxidant	Photocatalytic	Reference
1	Glucose stabilized AgNPs	Plasma reduction method	Size: 19.89 by XRD Shape: spherical	*E. coli*: 12 mm *S. auerus*: 18 mm	Antioxidant activity enhanced with rising amount of salt concentration by DPPH assay	82.3% MO degradation	Present study
2	fugus Trichoderma harzianum synthesis of extracellular AgNPs	Green synthesis	Size: 21.49 nm by DLS Structure: cubic	*S. auerus*: 14.6 mm, *B. subtilis*: 13.86 mm, *R. solanacearum*: 17.43, *E. coli*: 15.56 mm	Remarkable antioxidant Properties by DPPH and ferric reducing antioxidant power (FRAP) assay	-	[49]
3	AgNPs@SEE	biosynthesis	Size: 35–50 nm Shape: spherical Structure: cubic	*P. aeruginosa*, *E. faecalis*, *E. coli*, *S. aureus*, *P. mirabilis*, *A. baumannii* and *K. pneumonia*	-	MO degrade 95.89%	[62]
4	AgNPs) using Carissa opaca leaves	Green approach	Size: 8 nm	*E. coli*: 24 (±0.5 mm) *B. subtilis*: 20 (±0.8 mm)	Better antioxidant activity by DPPH assay	97% of MB in 50 min	[59]
5	chitosan/silver (CS/Ag) nanocomposite	bio-inspired method	Size: 23–78 nm Shape: spherical	*B. subtilis* and *E. coli*	Vitro antioxidant by DPPH	88% MB in 220 min	[20]
6	AgNPs	Biosynthesis method	Size: 30 nm Structure: microcrystalline	Highest antibacterial activity against *S. aureus* MTCC 902: (16 ± 0.904 mm)	Antioxidant by DPPH and H_2_O_2_ assay	-	[25]
7	AgNPs	Chemical route	Size: 4, 12 and 40 nm Shape: triangular	*E. coli* displays good antibacterial activity	-	-	[46,47]
8	AgNPs	Green synthesis	Size: 30 nm by SEM analysis	*E. coli*, *S. aureus*	Higher antioxidant activity using TFC, TPC, TAC, DPPH, FRAP, and IC50 assays	MO degradation in 20 min	[57]
9	AgNPs	Bio synthesis	Size: 39 ± 4 nm Shape: round plate-like Structure: FCC	Gram-negative low MIC as 5 ppm, Gram-positive 25 ppm	Potential antioxidant activity by using DPPH	-	[58]
10	AgNPs	Green synthesis by using stem (S), root (R), and leaf (L)	Size: S = 25.55 nm, R = 38.23 nm and L = 45.55 nm Shape: face hexagonal	-	-	MV, S, EMB and MO 30 min degradation	[7]

## 4. Conclusions

This paper reports on the BBD-optimized synthesis of glucose-stabilized AgNPs under response surface methodology (RSM) to optimize the AgNPs’ growth conditions. Discharge plasma-processed AgNPs were optimized through the manipulation of three different experimental parameters, namely, stabilizing agent concentration, metal salt concentration, and reaction time, as these parameters directly influence the effective growth of NPs. Quadratic equations acquired via the RSM aided in the selection of the effective experimental parameters for the desired (optimized) AgNP size (19.89 nm), which was confirmed by XRD analysis. The AgNPs that were prepared under optimized conditions showed a maximum absorbance peak at 430 nm, with a corresponding band gap of energy of 3.31 eV. The optimized experimental values were in good agreement with the predicted values. Therefore, the optimized synthesis of AgNPs can boost their antibacterial, antioxidant, and photocatalytic activity by improving their physiochemical properties. *E. coli* and *S. Aureus* strains were used to investigate the antibacterial efficacy of optimized AgNPs. Against both bacteria colonies, the optimal zone for the combination of stabilizing agents was measured at 12 mm and 18 mm, respectively. Meanwhile, the optimized AgNPs showed a maximum antioxidant activity of 81.2% when exposed to free radicals of DPPH. The photocatalytic efficiency of optimized AgNPs (run 4) for the degradation of MO under UV irradiation was observed to be approximately 82.3%. The enhanced antioxidant, antibacterial, and photocatalytic activity of optimized AgNPs make them a suitable material for catalytic activity. Future work may optimize various process parameters, including stirring time, the pH of the solution, and the concentration of dyes, for the optimized growth of AgNPs for the purpose of improving wastewater treatment.

## Figures and Tables

**Table 1:** Coded values with experimental and predicted responses obtained from a Box–Behnken experimental design.

Runs	A	B	C	Y = Size of AgNPs (nm) Experimental	Predicted
1	0	1	1	23.05	25.07
2	1	0	1	21.40	21.5
3	0	0	0	36.45	37
4	0	−1	1	19.89	20.38
5	−1	0	1	26.60	25.32
6	−1	0	−1	33.45	34
7	−1	1	0	25.33	24.5
8	1	−1	0	28.00	28.2
9	0	0	0	32.60	32.3
10	−1	−1	0	26.88	27
11	0	−1	−1	30.40	31.2
12	1	1	0	20.22	19.8
13	1	0	−1	25.33	24.5
14	0	0	0	25.55	22.36
15	0	1	−1	32.02	31.33

**Table 2:** Analysis of variance of quadratic model for AgNP synthesis.

Source	Sum of Squares	Degree of Freedom	Mean Square (Variance)	F-Value (Fisher’s Function)	*p*-Value (Level of Significance)	Determination of Coefficient R^2^
Model	306.989	9	34.10	16.039	0.002	0.96646
Residual	10.634	5	2.126			
Total	317.623	14				

**Table 3:** Variables levels selected for Box–Behnken design.

Variables	Main Effects	Coefficients	T-Value	*p*-Value Prob > F	Confidence Interval (C.I)
Intercept	461.962	230.981	4.441	0.000	100
*A*	–100.416	–50.208	–4.017	0.010	99.12
*B*	–2.002	–1.00097	–2.061	0.094	91.40
*C*	–5.791	2.895	1.762	0.138	86.06
*AB*	–0.084	–0.042	–0.869	0.424	58.14
*AC*	−1.597	–0.798	–4.560	0.005	99.343
*BC*	−0.00059	–0.00029	–0.026	0.980	1.99
*AA*	7.099	3.549	4.602	0.004	99.49
*BB*	0.040	0.0201	5.105	0.002	99.67
*CC*	0.448	0.224	3.309	0.021	98.14

**Table 4:** Mean particle size obtained by XRD and SEM analysis.

Sample Code	Average Particle Size (nm) by XRD Analysis	Average Particle Size (nm) by SEM Analysis
Run 4	19.89	82
Run 6	33.45	102

**Table 5:** The antibacterial activities and zones of inhibition of optimized AgNPs.

Samples	Size of AgNPs (nm)	Inhibition Zone (mm) *E. coli*	*S. aureus*
S1 (Run 1)	23.05	5	8
S2 (Run 2)	21.40	6	10
S3 (Run 12)	20.22	9	12
S4 (Run 4)	19.89	12	18

## Data Availability

Data supporting reported results is available and provided at reasonable request.

## References

[1] El-Naggar N.E.-A., Hussein M.H., El-Sawah A.A. (2017). Bio-fabrication of silver nanoparticles by phycocyanin, characterization, in vitro anticancer activity against breast cancer cell line and in vivo cytotxicity. Sci. Rep..

[2] Yazdi M.E.T., Darroudi M., Amiri M.S., Zarrinfar H., Hosseini H.A., Mashreghi M., Mozafarri H., Ghorbani A., Mousavi S.H. (2021). Antimycobacterial, Anticancer, Antioxidant and Photocatalytic Activity of Biosynthesized Silver Nanoparticles Using Berberis Integerrima. Iran. J. Sci. Technol. Trans. A Sci..

[3] Kakakhel M.A., Saif I., Ullah N., Faisal S., Anwar Z., Din S.Z.U. (2021). Waste Fruit Peel Mediated Synthesis of Silver Nanoparticles and Its Antibacterial Activity. BioNanoScience.

[4] Subramaniam P., Nisha K.M.J., Vanitha A., Kiruthika M.L., Sindhu P., Elesawy B.H., Brindhadevi K., Kalimuthu K. (2021). Synthesis of silver nanoparticles from wild and tissue cultured *Ceropegia juncea* plants and its antibacterial, anti-angiogenesis and cytotoxic activities. Appl. Nanosci..

[5] Loo Y.Y., Rukayadi Y., Nor-Khaizura M.-A., Kuan C.H., Chieng B.W., Nishibuchi M., Radu S. (2018). In Vitro Antimicrobial Activity of Green Synthesized Silver Nanoparticles Against Selected Gram-negative Foodborne Pathogens. Front. Microbiol..

[6] Keshari A.K., Srivastava R., Singh P., Yadav V.B., Nath G. (2018). Antioxidant and antibacterial activity of silver nanoparticles synthesized by Cestrum nocturnum. J. Ayurveda Integr. Med..

[7] Bhakya S., Muthukrishnan S., Sukumaran M., Muthukumar M., Kumar S.T., Rao M.V. (2015). Catalytic degradation of organic dyes using synthesized silver nanoparticles: A green approach. J. Bioremediat. Biodegred..

[8] Pinheiro S.K.D.P., Miguel T.B.A.R., Chaves M.D.M., Barros F.C.D.F., Farias C.P., de Moura T.A., Ferreira O.P., Paschoal A.R., Filho A.G.S., Miguel E.D.C. (2021). Silver nanoparticles (AgNPs) internalization and passage through the Lactuca sativa (Asteraceae) outer cell wall. Funct. Plant Biol..

[9] Sohal J.K., Saraf A., Shukla K.K. (2021). Silver Nanoparticles (AgNPs): Methods of Synthesis, Mechanism of Antimicrobial Action and Applications. Multidiscip. Res. Dev..

[10] Nguyen P.A., Phan H.P., Dang-Bao T., Nguyen V.M., Duong N.L., Huynh X.T., Vo P.P.T., Pham T.Y.L., Bui T.N.Q., Nguyen T. (2021). Sunlight irradiation-assisted green synthesis, characteristics and antibacterial activity of silver nanoparticles using the leaf extract of Jasminum subtriplinerve Blume. J. Plant Biochem. Biotechnol..

[11] Joshi P.G., More M.S., Bisht N., Phalswal P., Dhapte-Pawar V., Khanna P.K. (2021). Synthesis of biologically active silver nanoparticles using N-containing compounds: The dual role of semicarbazones. New J. Chem..

[12] Shepida M., Kuntyi O., Sozanskyi M., Sukhatskiy Y. (2021). Sonoelectrochemical Synthesis of Antibacterial Active Silver Nanoparticles in Rhamnolipid Solution. Adv. Mater. Sci. Eng..

[13] Hoang V.-T., Dinh N.X., Pham T.N., Hoang T.V., Tuan P.A., Huy T.Q., Le A.-T. (2021). Scalable Electrochemical Synthesis of Novel Biogenic Silver Nanoparticles and Its Application to High-Sensitive Detection of 4-Nitrophenol in Aqueous System. Adv. Polym. Technol..

[14] Taib T., Johan M.R., Basirun W.J. (2021). Plasmonic SERS active nanostructured Ag–SiO2 at optimum volume ratio synthesized via sol-gel technique. Phys. B Condens. Matter.

[15] Seku K., Hussaini S.S., Pejjai B., Al Balushi M.M.S., Dasari R., Golla N., Reddy G.B. (2020). A rapid microwave-assisted synthesis of silver nanoparticles using Ziziphus jujuba Mill fruit extract and their catalytic and antimicrobial properties. Chem. Pap..

[16] Sreelekha E., George B., Shyam A., Sajina N., Mathew B. (2021). A Comparative Study on the Synthesis, Characterization, and Antioxidant Activity of Green and Chemically Synthesized Silver Nanoparticles. BioNanoScience.

[17] Altaf N.U.H., Naz M.Y., Shukrullah S., Bhatti H.N., Irfan M., Alsaiari M.A., Rahman S., Niazi U.M., Glowacz A., Proniewska K. (2021). Statistically Optimized Production of Saccharides Stabilized Silver Nanoparticles Using Liquid–Plasma Reduction Approach for Antibacterial Treatment of Water. Materials.

[18] Fan J., Cheng Y., Sun M. (2020). Functionalized Gold Nanoparticles: Synthesis, Properties and Biomedical Applications. Chem. Rec..

[19] Jiang H., Chen Z., Cao H., Huang Y. (2012). Peroxidase-like activity of chitosan stabilized silver nanoparticles for visual and colorimetric detection of glucose. Analyst.

[20] Nandana C.N., Christeena M., Bharathi D. (2021). Synthesis and Characterization of Chitosan/Silver Nanocomposite Using Rutin for Antibacterial, Antioxidant and Photocatalytic Applications. J. Clust. Sci..

[21] Othman A.M., Elsayed M.A., Al-Balakocy N.G., Hassan M.M., Elshafei A.M. (2021). Biosynthesized silver nanoparticles by Aspergillus terreus NRRL265 for imparting durable antimicrobial finishing to polyester cotton blended fabrics: Statistical optimization, characterization, and antitumor activity evaluation. Biocatal. Agric. Biotechnol..

[22] Altaf N., Naz M., Shukrullah S., Bhatti H. (2021). Testing of photocatalytic potential of silver nanoparticles produced through nonthermal plasma reduction reaction and stabilized with saccharides. Main Group Chem..

[23] Skiba M., Vorobyova V. (2018). Green synthesis of silver nanoparticles using grape pomace extract prepared by plasma-chemical assisted extraction method. Mol. Cryst. Liq. Cryst..

[24] Singaram A.J.V., Ganesan N.D. (2021). Modeling the influence of extraction parameters on the yield and chemical characteristics of microwave extracted mango (*Mangifera indica* L.) peel pectin by response surface methodology. Prep. Biochem. Biotechnol..

[25] Husain S., Verma S.K., Hemlata, Azam M., Sardar M., Haq Q., Fatma T. (2021). Antibacterial efficacy of facile cyanobacterial silver nanoparticles inferred by antioxidant mechanism. Mater. Sci. Eng. C.

[26] Nyakundi E.O., Padmanabhan M.N. (2015). Green chemistry focus on optimization of silver nanoparticles using response surface methodology (RSM) and mosquitocidal activity: *Anopheles stephensi* (Diptera: Culicidae). Spectrochim. Acta Part A Mol. Biomol. Spectrosc..

[27] Zimmermann J.C. (2021). Probing Ultrafast Electron Dynamics in Helium Nanodroplets with Deep Learning Assisted Diffraction Imaging. Doctoral Dissertation.

[28] Yusof H.M., Mohamad R., Zaidan U.H., Rahman N.A.A. (2019). Microbial synthesis of zinc oxide nanoparticles and their potential application as an antimicrobial agent and a feed supplement in animal industry: A review. J. Anim. Sci. Biotechnol..

[29] Khan M., Khan S.T., Khan M., Adil S.F., Musarrat J., Al-Khedhairy A.A., Al-Warthan A., Siddiqui M.R.H., Alkhathlan H.Z. (2014). Antibacterial properties of silver nanoparticles synthesized using Pulicaria glutinosa plant extract as a green bioreductant. Int. J. Nanomed..

[30] Nadar S., Rathod V.K. (2016). Magnetic macromolecular cross linked enzyme aggregates (CLEAs) of glucoamylase. Enzym. Microb. Technol..

[31] Bezza F.A., Tichapondwa S.M., Chirwa E.M. (2020). Synthesis of biosurfactant stabilized silver nanoparticles, characterization and their potential application for bactericidal purposes. J. Hazard. Mater..

[32] Makvandi P., Wang C.Y., Zare E.N., Borzacchiello A., Niu L.N., Tay F.R. (2020). Metal-Based Nanomaterials in Biomedical Applications: Antimicrobial Activity and Cytotoxicity Aspects. Adv. Funct. Mater..

[33] Shahid M., Farooqi Z.H., Begum R., Naseem K., Ajmal M., Irfan A. (2018). Designed synthesis of silver nanoparticles in responsive polymeric system for their thermally tailored catalytic activity towards hydrogenation reaction. Korean J. Chem. Eng..

[34] Restrepo C.V., Villa C.C. (2021). Synthesis of silver nanoparticles, influence of capping agents, and dependence on size and shape: A review. Environ. Nanotechnol. Monit. Manag..

[35] Smiechowicz E., Niekraszewicz B., Kulpinski P. (2021). Optimisation of AgNP Synthesis in the Production and Modification of Antibacterial Cellulose Fibres. Materials.

[36] Wypij M., Świecimska M., Dahm H., Rai M., Golinska P. (2018). Controllable biosynthesis of silver nanoparticles using actinobacterial strains. Green Process. Synth..

[37] Boonyeun N., Rujiravanit R., Saito N. (2021). Plasma-Assisted Synthesis of Multicomponent Nanoparticles Containing Carbon, Tungsten Carbide and Silver as Multifunctional Filler for Polylactic Acid Composite Films. Polymers.

[38] Danaei M., Motaghi M.M., Naghmachi M., Amirmahani F., Moravej R. (2021). Green synthesis of silver nanoparticles (AgNPs) by filamentous algae extract: Comprehensive evaluation of antimicrobial and anti-biofilm effects against nosocomial pathogens. Biologia.

[39] Wang H., Zhang G., Mia R., Wang W., Xie L., Lü S., Mahmud S., Liu H. (2021). Bioreduction (Ag+ to Ag0) and stabilization of silver nanocatalyst using hyaluronate biopolymer for azo-contaminated wastewater treatment. J. Alloys Compd..

[40] Daniel M.-C., Astruc D. (2003). Gold Nanoparticles: Assembly, Supramolecular Chemistry, Quantum-Size-Related Properties, and Applications toward Biology, Catalysis, and Nanotechnology. Chem. Rev..

[41] Zayed M.F., Eisa W., El-Kousy S.M., Mleha W.K., Kamal N. (2019). Ficus retusa-stabilized gold and silver nanoparticles: Controlled synthesis, spectroscopic characterization, and sensing properties. Spectrochim. Acta Part A Mol. Biomol. Spectrosc..

[42] Das R., Nath S.S., Chakdar D., Gope G., Bhattacharjee R. (2010). Synthesis of silver nanoparticles and their optical properties. J. Exp. Nanosci..

[43] Jain S., Mehata M.S. (2017). Medicinal Plant Leaf Extract and Pure Flavonoid Mediated Green Synthesis of Silver Nanoparticles and their Enhanced Antibacterial Property. Sci. Rep..

[44] Zhao X., Li Q., Ma X., Quan F., Wang J., Xia Y. (2015). The preparation of alginate–AgNPs composite fiber with green approach and its antibacterial activity. J. Ind. Eng. Chem..

[45] Agnihotri S., Mukherji S., Mukherji S. (2014). Size-controlled silver nanoparticles synthesized over the range 5–100 nm using the same protocol and their antibacterial efficacy. RSC Adv..

[46] Van Dong P., Ha C.H., Binh L.T., Kasbohm J. (2012). Chemical synthesis and antibacterial activity of novel-shaped silver nanoparticles. Int. Nano Lett..

[47] Tak Y.K., Pal S., Naoghare P.K., Rangasamy S., Song J.M. (2015). Shape-dependent skin penetration of silver nanoparticles: Does it really matter?. Sci. Rep..

[48] Gunawan P., Guan C., Song X., Zhang Q., Leong S.S.J., Tang C., Chen Y., Chan-Park M.B., Chang M.W., Wang K. (2011). Hollow Fiber Membrane Decorated with Ag/MWNTs: Toward Effective Water Disinfection and Biofouling Control. ACS Nano.

[49] Konappa N., Udayashankar A., Dhamodaran N., Krishnamurthy S., Jagannath S., Uzma F., Pradeep C., De Britto S., Chowdappa S., Jogaiah S. (2021). Ameliorated Antibacterial and Antioxidant Properties by *Trichoderma harzianum* Mediated Green Synthesis of Silver Nanoparticles. Biomolecules.

[50] Betz A. (2020). Improving Analysis and Interpretation of Transcriptomics and Proteomics in Environmental Sciences. Doctoral Dissertation.

[51] Skiba M., Pivovarov A., Vorobyova V., Derkach T., Kurmakova I. (2019). Plasma-chemical formation of silver nanoparticles: The silver ions concentration effect on the particle size and their antimicrobial properties. J. Chem. Technol. Metall..

[52] Alavi M. (2022). Bacteria and fungi as major bio-sources to fabricate silver nanoparticles with antibacterial activities. Expert Rev. Anti-infective Ther..

[53] Sudarsan S., Shankar M.K., Motatis A.K.B., Shankar S., Krishnappa D., Mohan C., Rangappa K., Gupta V., Siddaiah C. (2021). Green Synthesis of Silver Nanoparticles by *Cytobacillus firmus* Isolated from the Stem Bark of *Terminalia arjuna* and Their Antimicrobial Activity. Biomolecules.

[54] Patil M.P., Seong Y.-A., Kim J.-O., Seo Y.B., Kim G.-D. (2021). Synthesis of silver nanoparticles using aqueous extract of Cuscuta japonica seeds and their antibacterial and antioxidant activities. Inorg. Chem. Commun..

[55] Dara P.K., Mahadevan R., Digita P.A., Visnuvinayagam S., Kumar L.R.G., Mathew S., Ravishankar C.N., Anandan R. (2020). Synthesis and biochemical characterization of silver nanoparticles grafted chitosan (Chi-Ag-NPs): In vitro studies on antioxidant and antibacterial applications. SN Appl. Sci..

[56] Kumar V., Singh D.K., Mohan S., Gundampati R.K., Hasan S.H. (2017). Photoinduced green synthesis of silver nanoparticles using aqueous extract of Physalis angulata and its antibacterial and antioxidant activity. J. Environ. Chem. Eng..

[57] Kandiah M., Chandrasekaran K.N. (2021). Green Synthesis of Silver Nanoparticles Using Catharanthus roseus Flower Extracts and the Determination of Their Antioxidant, Antimicrobial, and Photocatalytic Activity. J. Nanotechnol..

[58] Dilshad E., Bibi M., Sheikh N.A., Tamrin K.F., Mansoor Q., Maqbool Q., Nawaz M. (2020). Synthesis of Functional Silver Nanoparticles and Microparticles with Modifiers and Evaluation of Their Antimicrobial, Anticancer, and Antioxidant Activity. J. Funct. Biomater..

[59] Ullah I., Tahir K., Khan A.U., Albalawi K., Li B., El-Zahhar A.A., Jevtovic V., Al-Shehri H.S., Asghar B.H., Alghamdi M.M. (2022). Facile fabrication of Ag nanoparticles: An advanced material for antioxidant, infectious therapy and photocatalytic applications. Inorg. Chem. Commun..

[60] Frei M.S., Mondelli C., García-Muelas R., Morales-Vidal J., Philipp M., Safonova O.V., López N., Stewart J.A., Ferré D.C., Pérez-Ramírez J. (2021). Nanostructure of nickel-promoted indium oxide catalysts drives selectivity in CO_2_ hydrogenation. Nat. Commun..

[61] Al-Najar B., Younis A., Hazeem L., Sehar S., Rashdan S., Shaikh M.N., Albuflasa H., Hankins N.P. (2021). Thermally induced oxygen related defects in eco-friendly ZnFe2O4 nanoparticles for enhanced wastewater treatment efficiencies. Chemosphere.

[62] Hashemi Z., Mizwari Z.M., Mohammadi-Aghdam S., Mortazavi-Derazkola S., Ebrahimzadeh M.A. (2022). Sustainable green synthesis of silver nanoparticles using Sambucus ebulus phenolic extract (AgNPs@ SEE): Optimization and assessment of photocatalytic degradation of methyl orange and their in vitro antibacterial and anticancer activity. Arab. J. Chem..

[63] Lin W., Cao E., Zhang L., Xu X., Song Y., Liang W., Sun M. (2018). Electrically enhanced hot hole driven oxidation catalysis at the interface of a plasmon–exciton hybrid. Nanoscale.

